# Supplementary material for: Establishment and Characterization of Immortalized Miniature Pig Pancreatic Cell Lines Expressing Oncogenic K-RasG12D
Source: Int J Mol Sci. 2020 Nov 21;21(22):8820. doi: 10.3390/ijms21228820 (PMC7700231; doi:10.3390/ijms21228820)
Supplement: Supplementary file 1 [file ijms-21-08820-s001.pdf]

Supplementary information

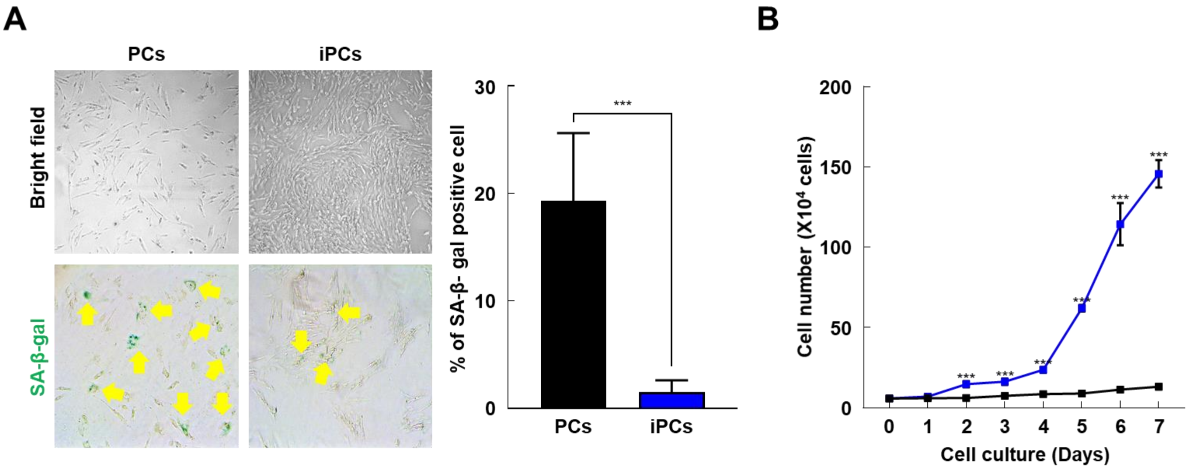

Figure S1. Differences in senescence and proliferation between PCs and iPCs. (**A**) Representative photograph of SA-β gal staining for 3 passage PCs and iPCs (left). Yellow arrows indicate SA-β gal positive cell. Graph of SA-β gal positive PC and iPC cell (Right). (**B**) Graph of cell count until 7 days. Magnification 100×. Data are presented as means ± standard deviation. *** $p < 0.001$, one-way ANOVA with Holm-Sidak multiple comparisons. All experiments were performed using at least three replicates, and the results are representative of three independent experiments.

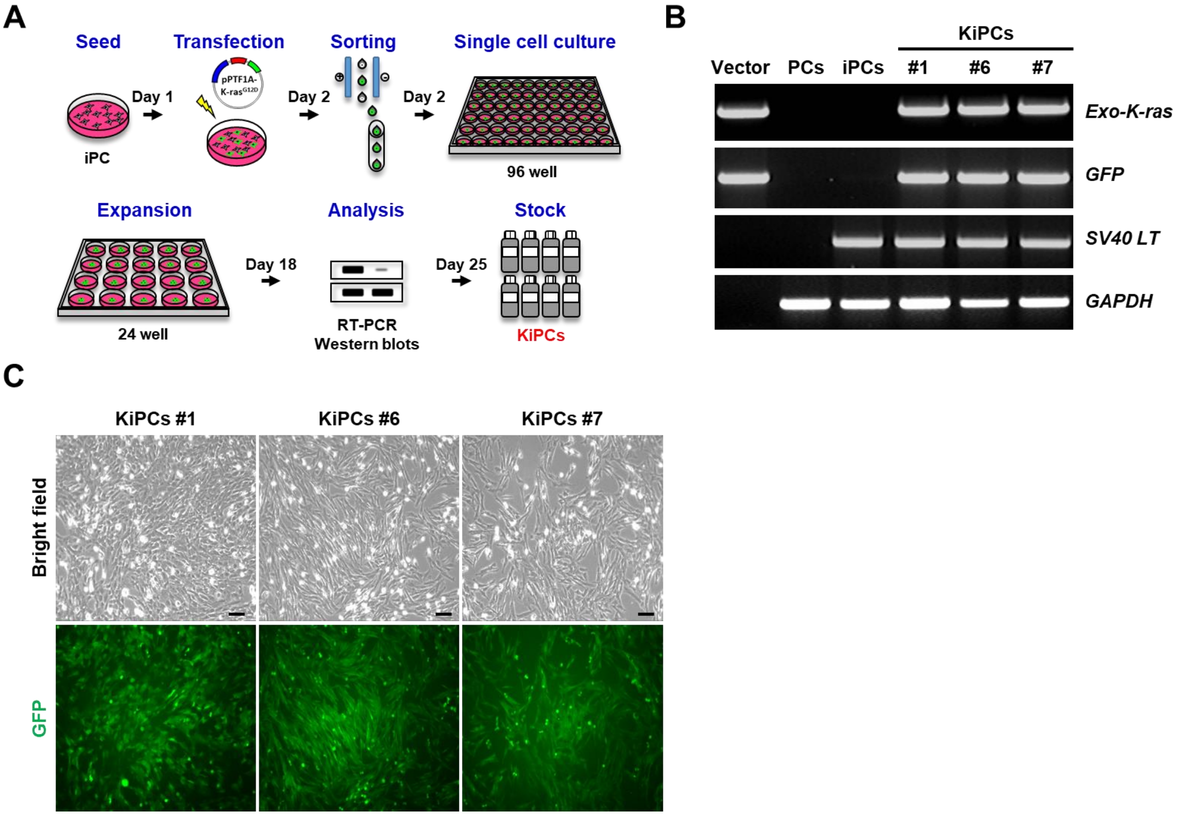

Figure S2. Establishment of KiPCs. (**A**) Schematic representation of the procedure for the establishment of KiPCs. (**B**) Semi-quantitative RT-PCR for K-ras, GFP, and SV40-LT in PCs, iPCs and KiPCs (#1, #6, and #7). (**C**) Representative immunofluorescence image of KiPCs. Magnification 100×.

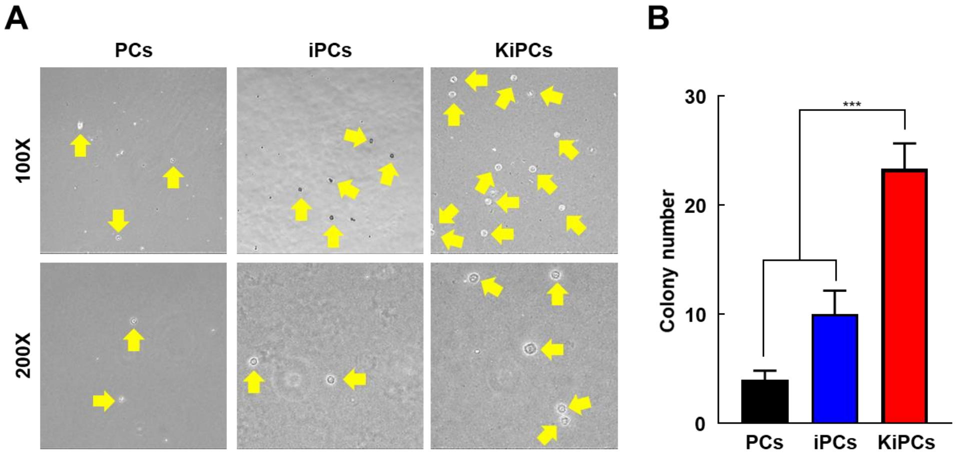

Figure S3. Anchorage-independent growth experiments cell growth ability (**A**) Representative image of soft-agar assay for PCs, iPCs and KiPCs. Yellow arrows indicate colony. (**B**) Graph of colony number. Average number values of colony were obtained from three independent filed. Magnification 100× or 200×. Data are presented as means ± standard deviation. *** $p < 0.001$, one-way ANOVA with Holm-Sidak multiple comparisons.

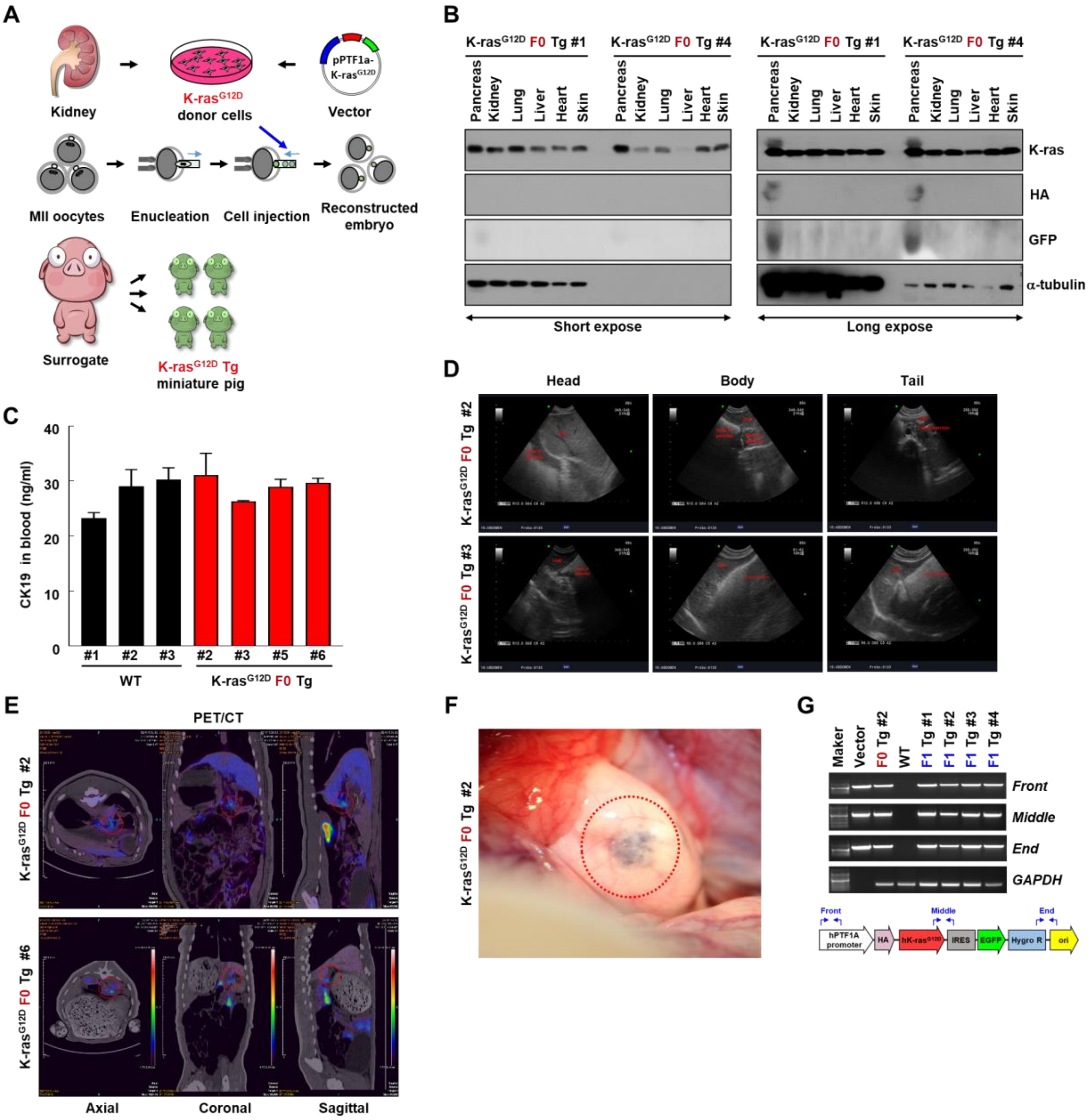

Figure S4. Generation of pancreatic specific-K-ras^{G12D} Tg miniature pigs and observation of abnormalities in pancreatic lesions. (**A**) Schematic diagram of generation for K-ras^{G12D} Tg miniature pigs. (**B**) Western blot analysis showed that the expression of exogenous K-ras^{G12D} only in pancreas of K-ras^{G12D} Tg miniature pigs. # indicated cloned miniature pig ID. (**C**) ELISA assay for pancreas specific maker of CK19 protein in blood from wild type (WT) (n = 3) and K-ras^{G12D} founders (F0) Tg (n = 4) after birth 10 months later. (**D**) Representative image of ultrasound examination for pancreatic abnormality from K-ras^{G12D} F0 Tg #2 and #3 after birth 5 months later. (**E**) Representative image of PET/CT for pancreatic abnormality from K-ras^{G12D} F0 Tg #2 and #6 after birth 12 months later. Colors illustrates level of FDG activity. Red dot circle indicates FDG activity in pancreas. (**F**) Visual inspection of pancreas from K-ras^{G12D} F0 Tg #2 after birth 15 months later. Red dot circle indicated abnormal location. (**G**) Genotyping of K-ras^{G12D} F0 Tg #2, WT, and offspring (F1) Tg (n = 4). Blue arrows represent PCR primers to intact integration of vector.

Table S1. Primer sequences for genotyping semi RT-PCR analysis.

	Gene Name	Accession No.	Primer Sequences (5′-3′)	Product Size
In vitro	*Exo K-ras*		F: GTGCCCGACTATGCCTCACT<br>R: TCCTGAGCCTGTTTTGTGTCT	437 bp
	Endo K-ras	XM_003126427.5	F: CCCAATTCCCTACCCACTGA<br>R: CATGACCACTGGGCTCACAT	489 bp
	EGFP		F: CCTGAAGTTCATCTGCACCA<br>R: TGCTCAGGTAGTGGTTGTCG	497 bp
	SV40-LT	NC_010447.5	F:ACAAGGGTTCGAGGACTGG<br>R: CGGCAGAGGAAAGAGAACAT	249 bp
	glyceraldehyde-3-phosphate dehydrogenase (GAPDH)	NC_010447.5	R: TTGCCTTCAGGTCAGGGAAT<br>R: TCAGCATTTTCCTGGCTGTC	479 bp
In vivo	*Front*		F: GCAATGAATTTCCCATGAGC<br>R: ACATGTGGGTAGGTGGAGGA	517 bp
	Middle		F: GGGTGTTGATGATGCCTTCT<br>R: TACGCTTGAGGAGAGCCATT	545 bp
	End		F: TCTACCAGAGCCTCGTGGAC<br>R: TTTTTGTGATGCTCGTCAGG	587 bp
	glyceraldehyde-3-phosphate dehydrogenase (GAPDH)	NC_010447.5	F: ACAAGGGTTCGAGGACTGG<br>R:CGGCAGAGGAAAGAGAACAT	249 bp

Table S2: Primer sequences for cell cycle regulated gene qRT-PCR analysis.

	Gene Name	Accession No.	Primer Sequences (5′-3′)	Product Size
Cell Cycle Inhibitor	*cyclin dependent kinase inhibitor 2A (CDKN2A, p14, p16)*	NM_213735.2	F: ATCCCGCAGACATTTCCAAC<br>R: ATTCTTCCCTGGGTTGCCTT	121 bp
	cyclin dependent kinase inhibitor 2B (CDKN2B, p15)	NM_214124	F:AGTGAGCGAGGAGGACAAGG<br>R:CTCCCGAAGTGGTTGAGTCC	144 bp
	cyclin dependent kinase inhibitor 3 (CDKN3, p16)	NM_214320.1	F: CTGCACCAGAGGGGAACTGT<br>R: CAGCAGCTGGCTATGTCAGG	126 bp
	cyclin dependent kinase inhibitor 2C (CDKN2C, p18)	XM_003127980.4	F: GCATACCGCCAGCAAAGTGG<br>R: TCGTTTCTGCGGTAGAGCCT	81 bp
	cyclin dependent kinase inhibitor 2D (CDKN2D, p19)	XM_005654859.2	F: TCCTGAACCGCTTTGGCAAG<br>R: TTGGGGCTGGCACCTTGTTT	94 bp
	cyclin dependent kinase inhibitor 1A (CDKN1A, p21)	XM_013977858.2	F: AGCCCCTCTGTCCAGTGAAA<br>R: CCAGAAAAGTGCAGGGGAAG	105 bp
	cyclin dependent kinase inhibitor 1B (CDKN1B, p27)	NM_214316.1	F: TCAGGCCAACTCAGAGGAACA<br>R:AGGAATCGTCTGTGGCAGGT	119 bp
Cyclin	*cyclin B1 (CCNB1)*	NM_001170768.1	F: AAGATGGAGGGGATCCAAAC<br>R: TGGCTCTCATGTTTCCAGTG	135 bp
	cyclin D1 (CCND1)	XM_021082686.1	F: CCCTCCGTGTCCTACTTCAA<br>R: AGGAAGCGGTCCAGGTAGTT	149 bp
	cyclin E1 (CCND1)	XM_005653265.2	F: AGCCACCTCCAGAACACCAC<br>R: AACGTGGCCCTCCTCAACTT	113 bp
Kinase	*cyclin dependent kinase 1 (CDK1)*	NM_001159304.2	F: CGCGGGATAATAAGCTGGGA<br>R: CATGGCTACCACTTGACCTGT	139 bp
	cyclin dependent kinase 2 (CDK2)	NM_001285465.1	F: CGGGCTGATTCGACTCGCTA<br>R: GGTGACGGGAGAAGTGGTGG	118 bp
	cyclin dependent kinase 4 (CDK4)	NM_001123097.1	F: ACCGTGTACAAAGCACGGGA<br>R:GCGCAGTAAGGCCACTTCAC	129 bp
Proliferation	*MYC proto-oncogene, bHLH transcription factor (c-MYC)*	NM_001005154.1	F: AGAGAAGCTGGCCTCCTACC<br>R: CAAGCTGGAGGTGGAGTAGC	91 bp
	proliferatin cell nuclear antigen (PCNA)	NM_001291925.1	F: TGGCTCCCAAGATCGAAGATGA<br>R: ATGTGCTGGCATCACCGAAG	101 bp
House Keeping	*glyceraldehyde-3-phosphate dehydrogenase (GAPDH)*	XM_021091114.1	F: CCCTGAGACACGATGGTGAA<br>R: GGAGGTCAATGAAGGGGTCA	127 BP

Table S3: Primer sequences for apoptosis regulated gene qRT-PCR analysis.

	Gene Name	Accession No.	Primer Sequences (5′-3′)	Product Size
Anti-apoptosis	apoptosis regulator (BCL2)	XM_021099593.1	F:GGAGGGGACACTCTTCTTCC<br>R:CTGGGCACAATTGGTAGCTT	189 bp
	BCL2 like 1 (BCL2L1)	NM_214285.1	F:AGGGCATTCAGTGACCTGAC<br>R:TGGATCCAAGGCTCTAGGTG	242 bp
Pro-apoptosis	BCL2 associated X, apoptosis regulator (BAX)	XM_00327290.5	F: AAGCGCATTGGAGATGAACT<br>R:CGATCTCGAAGGAAGTCCAG	251 bp
	BCL2 antagonist/killer 1 (BAK)	XM_013977773.2	F:CTAGAACCTAGCAGCACCAT<br>R:CGATCTTGGTGAAGTACTC	151 bp

Table 4: Primer sequences for pancreas specific marker qRT-PCR analysis.

	Gene Name	Accession No.	Primer Sequences (5′-3′)	Product Size
Ductal Maker	*carbonic anhydrase 2 (CA2)*	XM_001927805.2	F:CTAGAGACAGCGTCCAGCCGA<br>R: ATGGCAGCCAGAGACCAGTT	80 bp
	KRT19 (CK19)	XM_003131437.4	F:CAACGAGAAGCTCACCATGC<br>R:GGTACCAGTCGCGGATCTTC	116 bp
	SRY-box 9 (SOX9)	NM_213843.2	F: AAGAATAAGCCGCACGTCAA<br>R:CTCATTCAGCAGTCTCCAGAGTT	147 bp
Acinar Maker	*synaptophysin (SYP)*	XM_003135078.5	F:CAATGGGTCTTTGCCATCTT<br>R: GTACACTTCGTGCAGCCTGA	147 bp
	paired box 4 (PAX4)	XM_021078737.1	F: GACACGGTGAGGATCTGGTT<br>R:TGGGGAAGCACTTGGTAGAC	120 bp
	paired box 6 (PAX6)	NM_001244172.1	F: GTAGAACGCGGCTGTCAGAT<br>R:GGTTTGGTGTGTGAGAGCAA	136 bp
	pancreas associated transcrption factor 1a (PTF1A)	XM_003357758.4	F: CAGGCCCAGAAGGTCATCAT<br>R:AGGGGAGGGAGGCCATAAT	80 bp
	C1q and TNF related 1 (CTRP1)	XM_021066514.1	F:GTACGGCAAAACAGGCTCGG<br>R:GCGTAGTGGTTCTTGCACCG	102 bp
Endocrine Maker	*pancreatic alpha-amylase (AMY2A)*	XM_021090124.1	F:GTAGCAGGGTTCCGAATTGA<br>R: CAGGGAACCAGTTGGTGTTT	100 bp
	chromogranin A (CHGA)	NM_001164005.2	F:GGCAAGTCATTGCCCTCCCT<br>R:AGCGTGTCAGAGATGACCTCG	91 bp
	somatostatin (SST)	NM_001009583.1	F: CTGGGAAGCAGGAACTGG<br>R: GGACAAATCTTCAGGCTCCA	95 bp
	ISL LIM homeobox 1 (ISL1)	XM_003133934.5	F: CGAGAGCTGTACGTGCTTTG<br>R: CACGAAGTCGTTCTTGCTGA	118 bp

Table S5: Primer sequences for pancreatic cancer marker qRT-PCR analysis.

	Gene Name	Accession No.	Primer Sequence (5′-3′)	Product Size
Pancreatic Cancer Maker	*epithelial cell adhesion molecule (EPCAM)*	NM_214419	F: TGGGGAACTACTGGATCTGG<br>R: CAGCCTGTAGACCCTGCATT	92 bp
	CD44	XM_013994425	F: TGGAAGAGAGAAAGCCAAGC<br>R: GCCGTCATAAACTGGTCTGG	109 bp
	Prominin 1 (CD133)	XM_013978548.2	F: ATTGGTCTCTATAGGCAACATGG<br>R:TTCGTGCCGTTGGATGTGTT	70 bp
	MET proto-oncogene (c-met)	NM_001038008.1	F: CATTTTTATGGCCCCAACC<br>R: GATATTCATCACTGCGCACTTC	88 bp
